# Efficiency of HIV services in Nigeria: Determinants of unit cost variation of HIV counseling and testing and prevention of mother-to-child transmission interventions

**DOI:** 10.1371/journal.pone.0201706

**Published:** 2018-09-07

**Authors:** Sergio Bautista-Arredondo, Gina La Hera-Fuentes, David Contreras-Loya, Ada Kwan, S. Janae Van Buren, Ogbonna O. Amanze, Akinyemi Atobatele, Adedayo Adeyemi, Emmanuel Abatta, Kayode M. Ogungbemi, Sandra G. Sosa-Rubí

**Affiliations:** 1 National Institute of Public Health (INSP), Cuernavaca, Mexico; 2 School of Public Health, University of California, Berkeley, California, United States of America; 3 National Agency for the Control of AIDS (NACA), Abuja, Nigeria; 4 United States Agency for International Development (USAID), Abuja, Nigeria; 5 Centre for Infectious Disease Research and Evaluation, Abuja, Nigeria; 6 Federal Ministry of Health (FMoH), Abuja, Nigeria; Medizinische Universitat Wien, AUSTRIA

## Abstract

**Background:**

Like most countries with a substantial HIV burden, Nigeria continues to face challenges in reaching coverage targets of HIV services. A fundamental problem is stagnated funding in recent years. Improving efficiency is therefore paramount to effectively scale-up HIV services. In this study, we estimated the facility-level average costs (or *unit costs*) of HIV Counseling and Testing (HCT) and Prevention of Mother-to-Child Transmission (PMTCT) services and characterized determinants of *unit cost* variation. We investigated the role of service delivery modalities and the link between facility-level management practices and *unit cost* variability along both services’ cascades.

**Methods:**

We conducted a cross-sectional, observational, micro-costing study in Nigeria between December 2014 and May 2015 in 141 HCT, and 137 PMTCT facilities, respectively. We retrospectively collected relevant input quantities (personnel, supplies, utilities, capital, and training), input prices, and output data for the year 2013. Staff costs were adjusted using time-motion methods. We estimated the facility-level average cost per service along the HCT and PMTCT service cascades and analyzed their composition and variability. Through linear regressions analysis, we identified aspects of service delivery and management practices associated with unit costs variations.

**Results:**

The weighted average cost per HIV-positive client diagnosed through HCT services was US$130. The weighted average cost per HIV-positive woman on prophylaxis in PMTCT services was US$858. These weighted values are estimates of nationally representative unit costs in Nigeria. For HCT, the facility-level unit costs per client tested and per HIV-positive client diagnosed were US$30 and US$1,364, respectively; and the median unit costs were US$17 and US$245 respectively. For PMTCT, the facility-level unit costs per woman tested, per HIV-positive woman diagnosed, and per HIV-positive woman on prophylaxis were US$46, US$2,932, and US$3,647, respectively, and the median unit costs were US$24, US$1,013 and US$1,448, respectively. Variability in costs across facilities was principally explained by the number of patients, integration of HIV services, task shifting, and the level of care.

**Discussion:**

Our findings demonstrate variability in unit costs across facilities. We found evidence consistent with economies of scale and scope, and efficiency gains in facilities implementing task-shifting. Our results could inform program design by suggesting ways to improve resource allocation and efficiently scale-up the HIV response in Nigeria. Some of our findings might also be relevant for other settings.

## Introduction

To address the continued HIV burden the Nigerian government rolled out the 2010–2015 HIV/AIDS Strategic Plan and aimed to achieve 80% coverage of two primary pillars of the HIV response [1]–HIV Counseling and Testing (HCT) and Prevention of Mother-to-Child Transmission (PMTCT). However, despite significant progress, by 2014 Nigeria had only reached 45% coverage and the number of annual new infections exceeded 220,000, according to the most recent estimates [2]. Nigeria experiences the largest national HIV epidemic in the world, and by 2016 an estimated 3.2 million Nigerians were living with HIV [3].

Funding is a crucial constraint for Nigeria to expand and sustain the provision of HIV services. As other countries with generalized epidemics and high HIV prevalence, funding to support HIV programming relies heavily on international donors and has plateaued in recent years [4,5]. Therefore, to meaningfully scale-up the HIV response, Nigeria’s limited financial resources must be used optimally. To achieve this goal, improving efficiency in the implementation of HIV services is vital.

The Nigerian government has identified potential mechanisms to improve efficiency and increase access to HIV services. Among them are, decentralizing services from secondary and tertiary facilities to primary care clinics, integrating HCT services into routine health services, expanding demand generation activities, increasing task-shifting, and strengthening community mobilization [1]. However, the impact of these strategic recommendations on costs, efficiency, and quality of services is unclear.

Analyzing the costs of HIV services could help to identify the links between these approaches and efficiency, and to determine their relative importance. While previous costing studies have been conducted on HIV services in Nigeria, they have been limited in their scope, focusing solely on estimating the unit cost of services (the average cost per service delivered). However, in addition to assessing the unit cost of HIV services, identifying the determinants of unit cost variation can illuminate potential pathways to improve the performance of service delivery.

In this study, we applied micro-costing methods to a large and partially representative sample of facilities to assess costs and the determinants of cost variation of HCT and PMTCT services in Nigeria. In particular, we were interested in studying two aspects of service delivery and their association with efficiency. First, we explored the relationship between unit costs and service delivery models including the level of care (primary, secondary, or tertiary), integration of HIV services (HCT, PMTCT or ART provided in the same facility), task-shifting, and community involvement. Second, we explored the association between management practices and efficiency.

This study is part of a broader project, Optimizing the Response to Prevention and Treatment: HIV Efficiency in Nigeria (ORPTHEN). The objective of the study was to estimate and analyze average costs per services at the facility-level for the following HIV interventions: HIV counseling and testing (HCT), prevention of mother-to-child transmission (PMTCT), and antiretroviral treatment (ART) [6]. This paper focuses on the first two.

## Methods

For this study, we adapted to Nigeria the methods from a cross-sectional, micro-costing study conducted previously in four other African countries. Those methods have been described elsewhere [7]. Below we present those aspects relevant to this analysis and specific to Nigeria.

### Study sample

We applied multistage sampling first to select the 20 states with the highest HIV prevalence—we excluded three states due to security reasons—and subsequently to randomly sample facilities that provided at least one of the two interventions of interest (HCT or PMTCT). All levels of care (primary, secondary, and tertiary) were represented. Facilities were mostly integrated sites (offering both HCT and PMTCT services). At the time of data collection, 198 and 194 facilities in the sample provided HCT and PMTCT services, respectively; and they included teaching and referral hospitals, health centers, and maternal health clinics. HCT services in these facilities included both client-initiated and provider-initiated rapid HIV test algorithms (immunochromatographic assays). PMTCT services included HCT, routine clinical monitoring, and provision of ART and other drugs to pregnant women, mothers, and infants through Options A, B, and B+ (13.7%, 66.6%, and 19.7% of facilities, respectively).

Due to incomplete or missing information on outputs or staff composition, we excluded 46 HCT facilities and 43 PMTCT facilities from the sample. We eliminated an additional 11 HCT and 14 PMTCT facilities due to implausibly low or high unit costs. The final analytical sample includes 141 facilities offering HCT and 137 facilities providing PMTCT services (see S1 Fig in the supplemental materials).

Concerning implausible values of unit costs, we excluded facilities with fewer than 0.5 clients per day per provider or more than 100 clients per day per provider. However, we applied the former only to facilities which began providing services more than a year before data collection. We assumed that very early programs (less than a year old) require time to build demand for their services. On the other hand, HCT or PMTCT services with fewer than 0.5 clients per day per provider and more than one year providing those services were unlikely to be real or stable, and their inclusion in the sample could bias the results.

Given the relatively large proportion of excluded facilities, we performed an attrition analysis to assess the possible bias introduced by sample loss. We compared the subset of facilities excluded with those in the final analytical sample in terms of state, type of facility, level of care, total costs, total input costs, and total FTE. We present the results in S1 Table in supplementary materials.

### Data collection

Data collection took place between December 2014 and May 2015. Data on inputs, input prices, outputs, process quality, the competence of providers, staff’s time allocation, and facility characteristics—including management practices—were collected retrospectively from databases at the facility and district levels, program records, and monthly reports from the year 2013. Data were collected over two- to four-day time periods using standardized, pre-programmed, computer-based instruments by teams consisting of three trained surveyors. Facility-level questionnaires were administered to the facility’s manager in charge, consulting and validating the information on logs and records as much as possible. We collected data on input prices at the national level from the Ministry of Health. We also implemented a data quality assurance system which provided a mechanism for the research team to download the data on a weekly basis to assess completeness and quality of data [7].

### Measurement

#### Costs

We captured information on inputs used to produce HCT and PMTCT services. Namely personnel, recurrent inputs and services, capital (equipment and vehicles–see S2 Table for a comprehensive list of capital items), and training (opportunity costs of staff involved); and the prices of these elements–including salaries. We also measured outputs along the cascades of PMTCT and HCT services. For PMTCT, we measured the number of pregnant women tested, the number of HIV-positive pregnant women diagnosed, the number of HIV-positive women on treatment or prophylaxis, and the number of infants on prophylaxis. Women previously on ART were not included in the output measures. For HCT, we collected data on the number of people tested and the number of HIV-positive people diagnosed. All inputs and outputs were measured at the facility-level using standardized tools.

#### Time allocation

Time-motion methods were applied to estimate staff’s time allocation to each intervention. Time-motion is considered the gold standard approach to capture variation in time use across activities, and has been used in healthcare settings to assess optimal time allocation of staff, evaluate clinical workflow, and analyze potential improvements in efficiency [8–10]. In this study, observers randomly selected combinations of shifts, days of the week, and clinical providers (up to a maximum of six providers) to document the activities performed in blocks of three to four hours. The objective of this strategy was to achieve a representative sample of providers, days and hours reflecting the real variation of providers’ time allocation across interventions. Observers collected data on type and duration of activities such as direct service delivery, administrative work, meetings, and breaks. For each multitasking provider observed, time spent with patients for each intervention was assigned to that intervention whereas all other noncontact time was divided equally across the provider’s reported service areas. We used these observations to compute weights and applied them to multitasking staff in each facility. We allocated to either HCT or PMTCT the full salary of providers whose time was dedicated to either intervention exclusively.

#### Management

Management practices in for-profit firms are vital to the efficiency of production lines [11, 12]. Health providers, particularly those in public systems and not-for-profit private organizations, do not usually think about their work as a production process comparable to that in firms. However, not unlike private firms, health services are in fact produced by combining inputs—human and material—constrained by the underlying technology at their disposal. Different combinations of those inputs can result in varying levels of efficiency, and good management practices facilitate an efficient combination of inputs. Evidence suggests that management practices can positively affect health outcomes [12, 13]. In this study, we developed an instrument to capture management practices to assess the link between variations in management and unit costs.

We adapted the management framework from the ORPHEA project [7] to the Nigerian context. The ORPHEA questionnaire was based on work developed by Bloom *et al*. for private hospitals [13] and adapted for public facilities in a low- and middle-income country context.

S3 Table describes the specific questions used to measure each dimension of management included in the analysis. These dimensions capture a breadth of management practices that can affect clinic performance and were informed by literature [13] and previous data analysis [14]. The *performance-based funding* index describes the extent to which clinic performance indicators are linked to financial incentives (eg. number of clients served or quality indicators). *Individual incentives* summarize the different types of monetary, non-monetary and other in-kind incentives that staff can receive for individual-level good performance. Providing financial incentives for performance has been shown to impact the quantity and quality of services offered [15]. On the other hand, monetary incentives have been criticized on the basis that they appeal to extrinsic rather than intrinsic motivation, which is essential in health care [16]. However, evidence on the impact of these incentive schemes on the efficiency of HIV services is scarce [17]. The *sanctions* index measures the types of sanctions used to penalize poor individual performance. The *external supervision* index measures the amount of oversight received by the clinics. While excessive monitoring may negatively affect a facility’s ability to react swiftly to its needs and find solutions, external supervision may improve aspects of service delivery by providing staff guidance and feedback on performance. The *transparency* index measures the amount of information dissemination in which the clinic engages. Transparency may lead to less waste and more efficient use of resources, and can influence efficiency directly. The *community participation* index score measures how involved the community is in decision-making processes at the clinic (*e*.*g*. in the governing board) or in activities carried out by the clinics; facilities with more community involvement may be more effective at targeting services, engaging risk populations and retaining patients, thus influencing efficiency [13]. The *National Governance and Community Governance* indices measure the amount of involvement that the national government and community entities have over budgetary decisions, respectively. More oversight and support may lead to improved efficiency through better planning and more appropriate use of resources, or could cause more bureaucracy.

For each category of management, we constructed an index using additive scores. Their internal consistency was assessed using Cronbach’s alpha (alpha >0.80). We further validated the management indices by applying Principal Component Analysis (PCA) to all variables used in the six scores. We included eight variable scores in the models as binary variables for ease of interpretability. We defined the cut-off points according to their distribution: for performance-based funding and financial decisions by the community, we used the 75^th^ percentile, and for the remaining variables, we used the median. S2 Fig shows the distribution of the indices.

#### Service delivery characteristics

We measured other facility-level characteristics to investigate their associations with unit costs variations. S4 Table presents detailed definitions of these characteristics, the questions used to elicit them, and their specific values. We measured the scale of services as the total number of clients per intervention along both service cascades. For each facility, we captured the level of care, the types of HIV services provided, and site maturity, which measured the number of years providing each intervention. We also measured staff composition per intervention and created a task-shifting variable indicating that no physicians were involved in service delivery. Finally, we measured each provider’s years of experience on HIV services and took the average for each facility.

### Unit cost estimation

We estimated the total annual costs for each intervention applying a micro-costing approach from the perspective of service providers. Using quantities and prices of essential inputs as well as output data for both the HCT and PMTCT service cascades, we computed facility-level total costs by intervention, as follows:

$${TC}_{jk}=\sum_{i=1}^{i=4}{IC}_{ijk}$$

Where *TC*_*jk*_ denotes the total annual cost of intervention *j* (1 = HCT, 2 = PMTCT) at facility *k*. The term *IC*_*ijk*_ denotes the annual cost of input category *i* (1 = personnel, 2 = recurrent inputs and services, 3 = capital, and 4 = training), for intervention *j* at facility *k*.

We estimated the average cost per service per facility-intervention (or unit cost) for each step of the service cascade, as follows:

$${UC}_{{jk}_{l}}=\frac{{TC}_{jk}}{q_{jkl}}\;\mathrm{for}\;j=1,2,3,4$$

Where ${UC}_{{jk}_{l}}$ denotes the average cost (or unit cost) per output *l*, per intervention *j*, at facility *k*. TC*jk* indicates the total cost for intervention *j* at facility *k*; *q*_*jkl*_ denotes outputs along the cascade where *l* = 1 for clients tested, *l* = 2 for clients tested and HIV-positive, *l* = 3 for HIV-positive clients receiving ARV prophylaxis or treatment, and *l* = 4 for infants born to HIV-positive clients receiving NVP, with the latter two only defined for PMTCT. Given attrition along the service cascades, *q*_*j*11_ ≥ *q*_*j*12_ for HCT and *q*_*j*21_ ≥ *q*_*j*22_ ≥ *q*_*j*23_ ≥ *q*_*j*24_ for PMTCT.

For facilities providing more than one intervention, we apportioned shared inputs according to their contribution to total costs. Specifically, we weighted each category of shared input’s annual costs by the annual number of clients per intervention over the total yearly amount of outpatient clients in the facility. Finally, to allocate personnel costs across interventions, we applied weights derived from the time-motion study.

All cost data were converted from local currencies to United States Dollars (US$) using the daily mean exchange rate for the year 2013 (Nigeria: 150 Nigerian Naira–NGN). We report both unadjusted costs and costs adjusted for purchasing power parity (PPP) for non-tradable services, primarily staff salaries.

#### Estimation of weighted unit costs

While the variation of unit costs across facilities is the focus of the analysis in this paper, we also estimated weighted unit costs, which are better estimates of the national-level average costs per service, than the mean of facility unit costs. To determine the weighted unit costs, we take into account the relative contribution of services provided by each facility. We calculated the weighted unit cost as the sum of each facility-level unit cost multiplied by a non-negative weight. We defined the weight as the ratio between the number of annual clients in each facility and the total number of clients in the full sample, for each step in the cascade, as follows:

WUCjl=∑1nUCjkl*(OPjkl∑1nOPjkl)

Where *WUC*_*jl*_ denotes the weighted unit cost per output l, per intervention j. ${UC}_{{jk}_{l}}$ denotes the average cost (or unit cost) per output *l*, per intervention *j*, at facility *k*, summed from facility 1 to n. *OP*_*ijk*_ denotes the total number of clients per output *l*, per intervention *j*, at facility k, *and* $\sum_{1}^{n}{OP}_{jkl}$ denotes the total sample size per output, per intervention.

### Analysis of costs and the determinants of unit cost variation

#### Description of unit costs

First, we describe the unit cost per service along the cascade and the average composition of total costs, by input categories and level of care. Second, we show the dispersion of unit costs across facilities along the service cascades, by level of care. Third, we present the correlation between unit costs and scale, measured by the annual number of services provided.

#### Determinants of unit cost variation

We studied the efficiency of HIV services in Nigeria by analyzing the determinants of unit cost variation. We apply ordinary least squares (OLS) regression models, with robust standard errors to account for heteroskedasticity, as a function of outputs, facility-level characteristics, and the management indices, as follows:

$$ln{UC}_{ijk}=\alpha_{0}+\beta_{1}q_{ijk}+\beta_{1}q_{{ijk}^{2}+}\gamma_{1}m_{jk}+\gamma_{2}e_{k}+\gamma_{3}{lc}_{k}+\gamma_{4}c_{jk}+\gamma_{5}{ts}_{jk}+\delta_{1}{HIV}_{jk}+\delta_{2}{ART}_{k}+\theta X+\varepsilon$$

Where *UC*_*ijk*_ denotes the unit cost per output *i*, per intervention *j*, in facility *k–*with HCT (*j = 1*) and PMTCT (*j* = 2); and *i* = 1 for clients tested, *i* = 2 for HIV-positive clients identified, *i* = 3 for HIV-positive clients receiving ARV treatment or prophylaxis, and *i* = 4 for infants born to HIV-positive clients receiving NVP, with the latter two defined only for PMTCT.

On the right hand-side of the equation, *q*_*ijk*_ is the number of annual output *i* from intervention *j*, at facility *k*; *m*_*jk*_ measures the number of years since facilities began providing the intervention *j* (maturity) in facility *k*; *e*_*k*_ is the average number of years of staff experience providing HIV services in facility *k*; *lc*_*k*_ indicates the level of care of facility *k*; *c*_*jk*_ is the average competence score for intervention *j* in facility *k*; *ts*_*jk*_ is a binary variable indicating whether task shifting is used in providing the intervention *j* in facility *k*. As a measure of integration of HIV services we included two additional variables; *HIV*_*jk*_ measures the annual number of clients of intervention *j* in facility *k (with j = 1 when estimating UC*_*i2k*_, *and j = 2 when estimating UC*_*i1k*_*)*; and A*RT*_*k*_, a binary variable indicating whether the facility provided antiretroviral therapy (ART) or not. Finally, X is the vector of management indicators.

### Ethical clearance

The study was approved by the ethical review boards of the National Institute of Public Health, Mexico and the Nigerian Institute for Medical Research (NIMR).

## Results

### Average cost per service along the HCT and PMTCT service cascades

Table 1 shows sample sizes by intervention and level of care. The sample consisted of 141 HCT and 137 PMTCT facilities, of which roughly 22% were primary-level clinics, 62% were secondary hospitals, and 16% were tertiary hospitals. The average percentage of clients tested for HIV who were HIV-positive (positivity rate) was 11% in HCT services and 5% in PMTCT services. The proportion of HIV-positive patients who were on ARV treatment or prophylaxis was 77% in PMTCT services.

**Table 1:** HCT and PMTCT sample sites by facility type and cascade indicators.

	HCT		PMTCT	
**LEVEL OF CARE**	**N**	**%**	**N**	**%**
Primary	30	21	31	23
Secondary	90	64	82	60
Tertiary	21	15	24	17
Total	141	100	137	100
**Coverage rates**				
Average HIV positivity rates^a^	139	11	131	5
Average % of clients on ARV treatment or prophylaxis ^b^			120	77

Notes: HCT = HIV Counseliing and Testing

PMTCT = Prevention of Mother-to-Child Transmission

^a^ Defined as the percentage of HIV-positive clients with respect to all clients tested

^b^ Defined as the percentage of clients on ARV treatment or prophylaxis with respect to all HIV-positive clients

Table 2 displays the average annual number of clients per facility along the service cascades in 2013. On average, facilities tested 2,940 individuals per year and diagnosed 307 HIV-positive individuals through HCT services. The PMTCT services tested on average 1,577 women per year and diagnosed 59 HIV-positive women.

**Table 2:** Average number of clients per facility along the HCT and PMTCT service cascades.

	n	Mean	SD	Median
**HCT**				
Annual number of clients tested	141	2,940	3,197	1,799
Annual number of HIV-positive clients diagnosed	139	307	400	135
**PMTCT**				
Annual number of women tested	137	1,577	1,857	884
Annual number of HIV-positive women diagnosed	131	59	113	22
Annual number of HIV-positive women on ARV treatment or prophylaxis	120	36	50	17
Annual number of infants on NVP prophylaxis	97	29	36	16

Notes: n = sample size; SD = standard deviation

HCT = HIV Counseling and Testing

PMTCT = Prevention of Mother-to-Child Transmission

Table 3 displays the summary of unit costs for each step of the HCT and PMTCT service cascades. The weighted average cost per HIV-positive client diagnosed was US$130 and US$858, in HCT and PMTCT services, respectively.

**Table 3:** Unit costs along the HCT and PMTCT service cascades (US$).

	n^a^	Weighted	Mean	SD	Median
		Mean^b^			
**HCT unit costs**					
Average cost per client tested	141	13	30	41	17
Average cost per HIV-positive clients diagnosed	139	130	1,364	4,738	245
**HCT unit cost (PPP)** ^c^					
Average cost per client tested	141	22	50	73	29
Average cost per HIV-positive clients diagnosed	139	214	2,223	7,606	399
**PMTCT unit costs**					
Average cost per women tested	137	19	46	69	24
Average cost per HIV-positive women diagnosed	131	507	2,932	4,622	1,013
Average cost per HIV-positive women on ARV treatment or prophylaxis	120	858	3,647	5,479	1,448
Average cost per infants on NVP prophylaxis	97	1,133	4,242	6,230	1,700
**PMTCT unit costs (PPP)** ^c^					
Average cost per women tested	137	29	74	120	36
Average cost per HIV-positive women diagnosed	131	794	4,803	7,964	1,425
Average cost per HIV-positive women on ARV treatment or prophylaxis	120	1,341	5,933	9,389	2,201
Average cost per infants on NVP prophylaxis	97	1,761	6,819	10,515	2,497

n, sample size; SD, standard deviation; HCT, HIV counseling and testing; PMTCT, prevention of mother-to-child transmission; Unit costs in 2013 US Dollars.

^a^ Different sample sizes along the cascades are due to missing values.

^b^ Weighted mean represents a nationally representative average value, considering the relative contribution of each facility in terms of its patient volume. It was calculated as the sum of each data point multiplied by a non-negative weight (defined as the number of annual HCT or PMTCT clients in each step of the cascade, divided by the total number of annual HCT or PMTCT clients in each step of the cascade, in the full sample).

^c^ Purchase power parity.

The average unit cost per HCT client tested was US$30, while the average unit cost per HIV-positive client was US$1,364. The equivalent figures in PMTCT services were US$46 and US$2,932, respectively. Average and median costs per unit of output varied substantially in both interventions, which implies a significant variation in unit costs across facilities.

**Fig 1:**
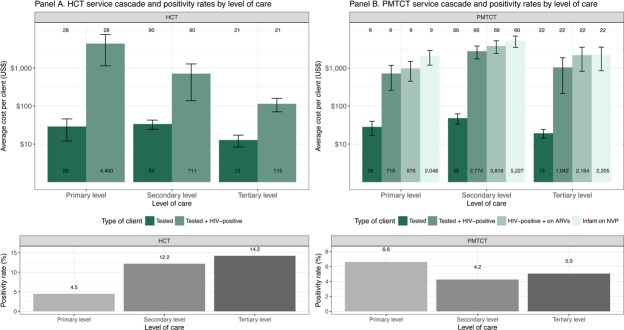
Unit cost per client across the service cascade and positivity rates of HCT and PMTCT services. Note: Labels inside the bars represent actual figures; labels above the bars represent the sample size.

Fig 1 shows HCT and PMTCT average unit costs per client along the service cascades, by level of care. The bottom panels of the Figure present the HIV positivity rates—measured at the facility-level and defined as the proportion of tests with HIV-positive results. In the case of HCT, the unit cost per person tested is not significantly different by level of care, albeit slightly lower in tertiary facilities. The cost per person diagnosed does vary substantially across levels of care, with unit costs significantly decreasing as level increases. This difference is associated with positivity rates, with lower positivity rates at the primary level. In the case of PMTCT services, we found the lowest HIV positivity rates in the secondary level, which resulted in significantly higher costs per service, compared to primary and tertiary facilities.

**Fig 2:**
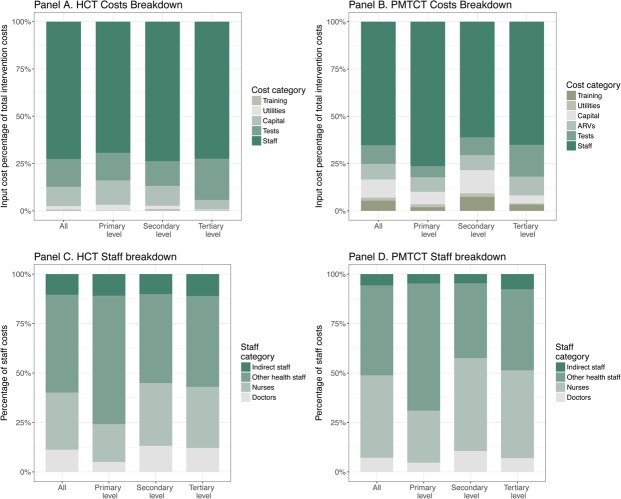
Breakdown of HCT and PMTCT costs, by level of care.

We further explored the composition of PMTCT and HCT costs in Nigeria. Fig 2 presents the breakdown of the total costs into the main input categories; overall and by level of care. In the case of HCT (Panel A), the staff category is by far the largest component, accounting for roughly 70% of the total costs. HIV test kits (15%) and capital goods (10%) are the second and third cost drivers, respectively. Utilities contributed 2% to the total costs. The distribution is similar across levels of care.

Regarding PMTCT services (Panel B), the staff category accounted by two-thirds of the total costs (65%), followed by HIV test kits (10%), capital costs (10%), antiretroviral drugs (8%), training (5%), and utilities (2%). The distribution was not significantly different across levels of care, except for the primary level, where staff represents a larger share of the total costs (76%).

In panels C and D, we further break down the staff category into four cadre classes: doctors, nurses, other health staff (counselors, nutritionists, etc.), and indirect staff (administrative, maintenance, etc.). Other health staff accounted for roughly 50% of the total staff costs in HCT, followed by nurses (30%), doctors (10%) and indirect staff (10%). In PMTCT, other health staff and nurses accounted for 46% and 42%, respectively, doctors 7%, and indirect staff 5%.

### Variation of unit costs along the HCT and PMTCT service cascades

**Fig 3:**
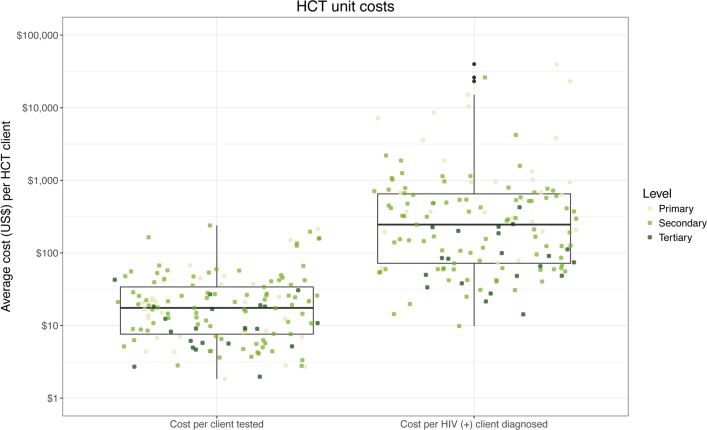
HCT average costs by cascade stage and level of care. Note: Lines inside the box indicate the median of the distribution; boxes depict the inter-quartile range (IQR); whiskers extend to 1.5 times the IQR.

In Fig 3 we present the dispersion of HCT unit costs for two steps of the service cascade, by level of care. Each dot represents a facility and the vertical axis measures the unit cost. The results show the degree of variation in unit costs among facilities, which ranged across three and four orders of magnitude—higher in the cost per HIV-positive case diagnosed. Secondly, we observe facilities from the three levels of care across all ranges of costs.

**Fig 4:**
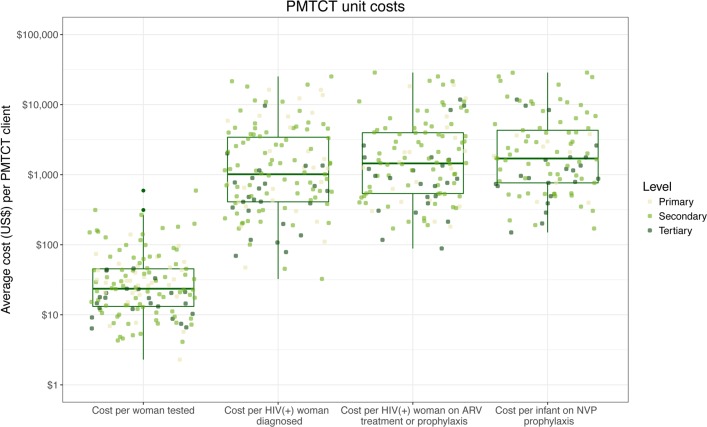
PMTCT average costs by cascade stage and level of care. Note: Lines inside the box indicate the median of the distribution; boxes depict the inter-quartile range (IQR); whiskers extend to 1.5 times the IQR.

The unit costs of PMTCT services also varied considerably among facilities with differences of two and three orders of magnitude for the same service across facilities (see Fig 4). In the case of PMTCT, we observe four outputs along the service cascade, and the PMTCT findings are consistent with those described above for HCT.

**Fig 5:**
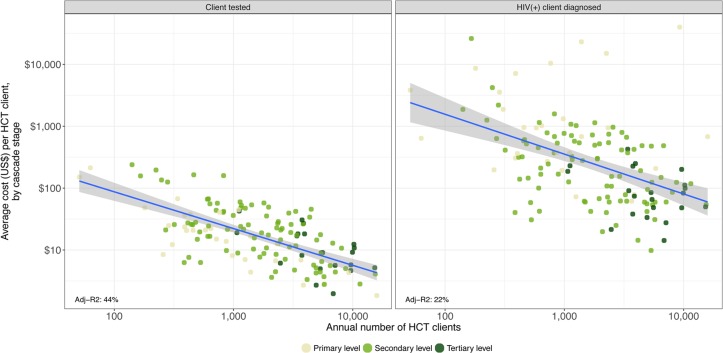
Relationship between HCT unit costs and scale, by cascade stage.

The volume of services is a critical factor in determining the efficiency of HCT and PMTCT services. Fig 5 displays the relationship between the facility-level unit costs of HCT services (in the vertical axis) and the volume of clients or scale (in the horizontal axis). Again, each dot represents a facility. Overall, there is a negative relationship between costs and scale; a greater volume of clients is associated with lower unit costs, with scale accounting for 44% of the variability of the average cost per client tested and 22% of the variation in the average cost per HIV-positive client identified.

**Fig 6:**
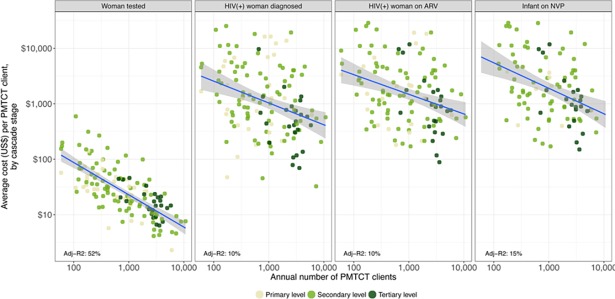
Relationship between PMTCT unit cost and scale, by cascade stage.

We also found a negative correlation between the volume of PMTCT clients per facility and unit costs (see Fig 6). About 52% of the variability of the PMTCT unit costs by client tested was explained by scale.

### Determinants of unit costs variation along the HCT and PMTCT service cascades

**Fig 7:**
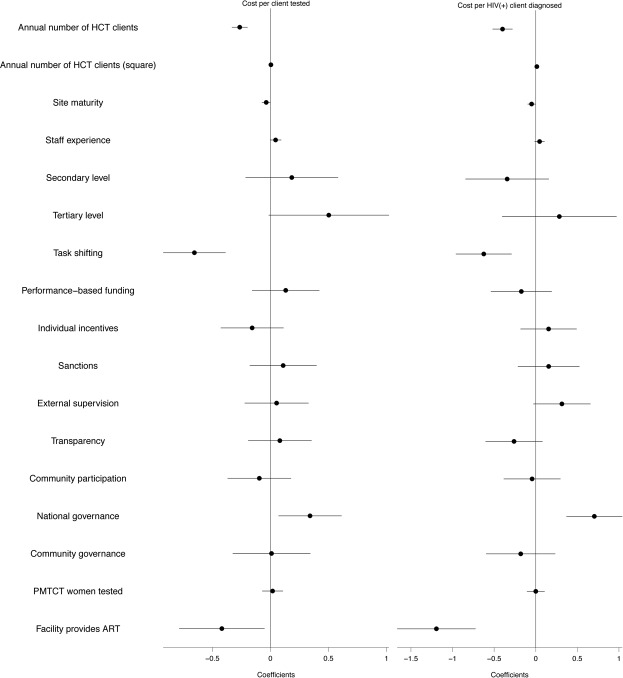
HCT regression models along the service cascade.

We explored determinants of unit cost variation, including scale, scope, task-shifting, and managerial practices. S5 Table shows the results of two specifications of the regression model for HCT unit costs—per client tested and per HIV-positive client diagnosed—against the volume of clients, other service delivery characteristics, and dimensions of management. The difference between the two specifications is the inclusion of quality in Specification 2, measured by the competence score (see S1 File for details on this variable). Fig 7 graphically presents the results of the first specification for both models.

As shown in Fig 7, everything else being equal, we found a negative association between the number of HCT clients and *unit costs* in both steps of the cascade, which is consistent with economies of scale. The square term of the number of HCT clients was positive and significant, which indicates a decreasing rate of economies of scale. The level of service provision was not significantly associated with unit costs once we controlled for other characteristics—except for the unit cost per client tested at the tertiary level. We observed an association between task-shifting and lower unit costs in both steps of the cascade.

Regarding management characteristics, we found associations between external supervision and the involvement of the government in financial decisions, with higher unit costs per person tested. Providing individual incentives was negatively associated with HCT unit costs. Finally, we found that everything else being equal, unit costs of HCT services were lower in facilities that also provided ART services (a measure of integration of services).

**Fig 8:**
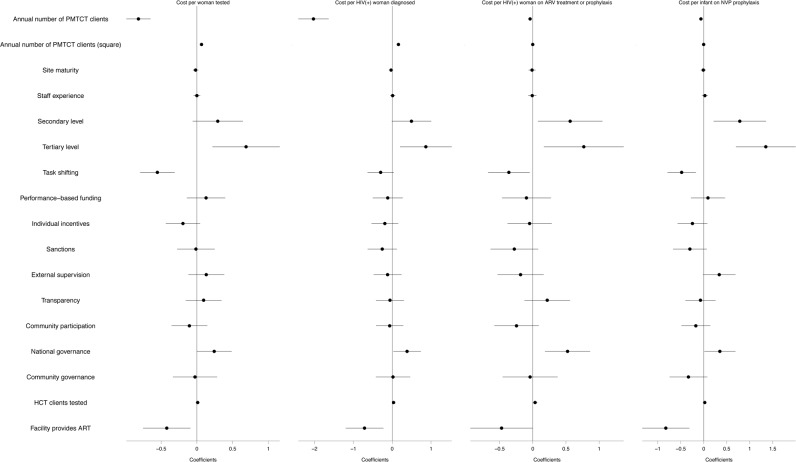
PMTCT regression models along the service cascade.

S6 Table presents the results of the regression models for unit costs of PMTCT services, along four indicators in the service cascade, and Fig 8 displays the results graphically.

As shown in Fig 8, everything else being equal, we found a negative association between the number of PMTCT clients and unit costs in all steps of the cascade, although the strength of this association decreases as we move further. The square term of the number of PMTCT clients was positive and significant. These results, as in the case of HCT, are consistent with economies of scale at a decreasing rate. Tertiary-level facilities were associated with higher unit costs. We found a statistical association between task-shifting and lower unit costs in all steps of the cascade.

Regarding management, we found an association between involvement of the government in financial decisions and higher costs per PMTCT client. Performance-based funding was associated with higher unit costs, although this relationship was statistically significant only in the first step. We observed a negative association between PMTCT unit costs and individual incentives and community involvement. Similar to HCT, facilities with ART services showed lower unit costs of PMTCT services. Finally, we found a positive correlation between competence and PMTCT unit costs (S5 Table).

## Discussion

In a large and representative sample of facilities in Nigeria, we estimated the costs of HCT and PMTCT services cascades in Nigeria. The weighted average cost per HIV-positive client diagnosed in HCT services was US$130. The weighted average cost per HIV-positive woman on prophylaxis in PMTCT services was US$858. Weighted unit costs are estimates of the nationally representative costs in Nigeria given that we compute them accounting for each facility’s contribution to the national average based on their volume of patients. The estimated facility-level unit cost per client tested and per HIV-positive client diagnosed were US$30 and US$1,364 for HCT and US$46 and US$2,932 for PMTCT, respectively. The analysis on the composition of costs revealed that for both HCT and PMTCT services, costs are mainly driven by staff followed by HIV tests and ARV drugs, the latter only in the case of PMTCT. We found that the composition of costs was similar across levels of care. Other health staff and nurses categories accounted for the highest proportion of staff costs for both HCT and PMTCT.

Unit costs increase along the cascade of HCT and PMTCT services, which is directly determined by two factors: positivity rates and attrition rates. For example, in the case of PMTCT, the gap between the cost per woman tested and the cost per HIV-positive woman diagnosed depends on the number of women that must be tested to find one HIV-positive case; lower positivity rates determine a higher cost per HIV-positive case. The gap between the cost per HIV-positive woman diagnosed and the cost per woman on ARV prophylaxis or treatment is determined by the proportion of women lost between these two steps in the cascade; a larger gap indicates more women lost proportionally. Our results suggest that secondary level facilities have more attrition than primary and tertiary ones, which may be an aspect of service delivery worth exploring in future studies.

We also found variation in unit costs across levels of care. In the case of HCT services, the unit cost per HIV-positive client diagnosed decreases as the level of care increases. In PMTCT services, unit costs are higher among secondary level facilities than primary and tertiary, which is explained by HIV positivity rates in these facilities.

The results of this study demonstrate variation in unit costs across facilities; we found differences of three to four orders of magnitude in costs for the same services across levels of care and along the service cascades. A large proportion of this variation was explained by scale, particularly in the first step of both cascades.

However, even after controlling for scale, a substantial level of heterogeneity in unit costs persisted. Overall, we found that efficiency was linked to service delivery characteristics and management practices. At the national level, we observed evidence of economies of scope. We found that integration of HIV services, measured by the number of PMTCT or HCT clients tested and by the presence of ART services, seemed to be efficient. Facilities providing integrated HCT and PMTCT services, or ART services, were less costly. We also found evidence that task-shifting consistently predicts lower unit costs across the service cascades of both interventions. At the facility level, we found statistically significant associations between unit costs and management practices. Government involvement in financial decisions, external supervision, and performance-based funding, were practices associated with higher costs, whereas individual incentives and community involvement were associated with lower unit costs. Quality (competence score, which we described in S1 File) was not significantly associated with unit costs.

Our cost estimates were captured using a representative sample of facilities and were found to be comparable to previous estimates[18, 19]. In an earlier study, Aliyu et al. [20] reported an average cost per HCT client of US$7.40, with US$18.50 in tertiary level facilities and US$6.30 in secondary level facilities. In comparison, we found a weighted mean of US$13. However, our study includes a sample of 141 facilities from first, second and third levels of care; in contrast, their research consists of nine facilities from the secondary and tertiary levels of care. Furthermore, evidence of high variation in unit costs at the facility-level is consistent with other studies [21, 22]. Similar to previous studies, we observed evidence of economies of scale [23,24]. However, we also found evidence consistent with economies of scope—integrating HIV services—for which previous studies have found inconclusive evidence [25].

Our study contributes knowledge on the determinants of unit costs variation. We focused on aspects of service delivery. We found that scale, level of care, task-shifting, and community involvement in the provision of services, were aspects statistically associated with variations in unit costs of HIV programs. Although more research on the cost-effectiveness of these modalities is needed, our results suggest a potential role for them in improving efficiency.

To our knowledge, this study—along with the larger ORPHEA study—is the first to assess the role of management practices on the variation in costs of HIV services. For example, while limited previous research has examined the impact of supervision on the quality of services [26, 27] and other management practices [13], no studies have assessed their effects on efficiency. Similarly, little or no evidence exists on the impact of task-shifting on unit costs.

Some limitations should be considered when interpreting our findings. The cross-sectional design of the study does not allow us to identify causality. Our estimates relied on administrative records at the facility-level, which in some cases were incomplete and, in all cases, are prone to measurement error. Our costs estimates rely on the results of time-motion measurements, which is considered the gold-standard method to estimate time allocation across tasks and processes. However, this method is also vulnerable to measurement error resulting from at least two potential sources. One is the possibility that the sample of providers/days/hours was not representative of time allocation throughout the year. The direction of the bias resulting from this potential measurement error is difficult to predict. The second limitation is the Hawthorne effect. Providers may have changed their behavior while being observed, leading to an overestimation of staff costs, if providers chose to use more time than usual working on HCT or PMTCT services knowing we were assessing these interventions. However, this potential bias would only affect multitasking staff. Finally, time allocation is not just a function of the providers’ decisions but also depends on service demand by clients, thereby further reducing this potential bias.

We adapted the instruments to capture management practices from a previous study. We examined eight mutually exclusive dimensions of management. However, it is possible that we did not measure other aspects of management potentially relevant for the production of PMTCT and HCT services. Quality of services was assessed through provider’s competence on processes and did not include other dimensions of quality.

Finally, due to missing data and implausible values in unit costs, we lost approximately 30% of facilities from the original sample. We explored the implications of this level of attrition comparing the subset of facilities lost with those in the analytical sample. First- and second-level facilities accounted for most of the facilities lost due to missing values. We found statistical differences in the distributions of HCT facilities by level of care and state. In the case of PMTCT, we observed statistical differences in the distribution by state. Nevertheless, we did not find differences in total costs, capital costs, staff distribution (full-time equivalents by type of provider), and ownership. And these results hold for both interventions, suggesting that our main findings and conclusions in the paper are unbiased (S1 Table).

Despite these limitations, our study provides sound estimates of unit costs of HCT and PMTCT services in Nigeria. Considering the sample size, sampling strategy, and aspects of service provision analyzed, this is the most comprehensive study on the efficiency of HCT and PMTCT services in Nigeria to date. Our study adds to the literature on the efficiency of HIV programs and illuminates determinants of cost variation. Our results could be used to inform program design to optimize the use of resources and sustain coverage of HIV services.

## Supporting information

S1 File Quality measurement. (DOCX)

S1 Fig Analytical sample. (TIF)

S2 Fig Management indices scores per intervention. (TIF)

S1 Table Comparison of facilities excluded with the final analytical sample. (XLSX)

S2 Table Description of elements included in capital. (XLSX)

S3 Table Measurement of management. (XLSX)

S4 Table Measurement of other facility-level characteristics. (XLSX)

S5 Table HTC regression models. Dependent variable is the natural logarithm of unit cost.
(XLSX)

S6 Table PMTCT regression models. Dependent variable is the natural logarithm of unit cost.
(XLSX)
