# Supplementary material for: Efficiency of HIV services in Nigeria: Determinants of unit cost variation of HIV counseling and testing and prevention of mother-to-child transmission interventions
Source: PLoS One. 2018 Sep 7;13(9):e0201706. doi: 10.1371/journal.pone.0201706 (PMC6128456; doi:10.1371/journal.pone.0201706)
Supplement: S1 File — (DOCX) [file pone.0201706.s001.docx]

**S1 File. Quality measurement**

Process quality was measured from the perspective of providers using clinical vignettes. Providers are asked to indicate the processes and procedures they would perform when presented hypothetical cases. The responses are scored against a set of correct answers, determined by a gold standard – in our case, official clinical guidelines valid in Nigeria at the time of survey application.^[[1]](#footnote-1),^^[[2]](#footnote-2)^ Vignettes can capture process quality and determine providers’ level of competence by identifying specific areas where providers do and do not indicate appropriate procedures in the clinical vignette. In studies in low- and middle-income countries, vignettes have been shown to capture variations in clinical practice^[[3]](#footnote-3)^ and have acquired a broader role in evaluating quality of care in low- and middle-income countries as well as in the U.S.^[[4]](#footnote-4)^ Quality scores obtained via vignettes have consistently demonstrated close scores to those obtained by the gold standard of Standardized Patients, a simulation exercise widely used to measure quality of care that involves actors and physicians^[[5]](#footnote-5),^^[[6]](#footnote-6)^ In this study, vignettes were applied to up to five providers per facility. For PMTCT, the clinical vignettes included questions related to initial assessments and care recommendations; and for HCT, the questions were related to pre-counseling, pre-testing, testing and post-test counseling^[[7]](#footnote-7)^ The quality scores were estimated by adding the number of correct answers and computing the proportion of correct answers with respect to the total number of items.

1. National Guidelines for HIV Counselling and Testing. Government of Nigeria: Federal Ministry of Health; 2011. [↑](#footnote-ref-1)
2. National Guidelines for Prevention of Mother-to-Child Transmission of HIV (PMTCT). Government of Nigeria: Federal Ministry of Health; Fourth Edition, 2010 [↑](#footnote-ref-2)
3. Das J, Gertler PJ. Variations in practice quality in five low-income countries: a conceptual overview. Health affairs. 2007 May 1;26(3):w296-309. [↑](#footnote-ref-3)
4. Aung T, Montagu D, Schlein K, Khine TM, McFarland W. Validation of a new method for testing provider clinical quality in rural settings in low-and middle-income countries: the observed simulated patient. PLoS One. 2012 Jan 23;7(1):e30196. [↑](#footnote-ref-4)
5. Peabody JW, Luck J, Glassman P, Dresselhaus TR, Lee M. Comparison of vignettes, standardized patients, and chart abstraction: a prospective validation study of 3 methods for measuring quality. Jama. 2000 Apr 5;283(13):1715-22. [↑](#footnote-ref-5)
6. Peabody, J.W., Luck, J., Glassman, P., Jain, S., Hansen, J., Spell, M. and Lee, M., 2004. Measuring the quality of physician practice by using clinical vignettes: a prospective validation study. *Annals of internal medicine*, *141*(10), pp.771-780. [↑](#footnote-ref-6)
7. Bautista-Arredondo S, Sosa-Rubi SG, Opuni M, Kwan A, Chaumont C, Coetzee J, et al. Assessing cost and technical efficiency of HIV prevention interventions in sub-Saharan Africa: the ORPHEA study design and methods. BMC Health Serv Res. 2014;14:599. [↑](#footnote-ref-7)
