# Supplementary figures and images for: Efficiency of HIV services in Nigeria: Determinants of unit cost variation of HIV counseling and testing and prevention of mother-to-child transmission interventions
Source: PLoS One. 2018 Sep 7;13(9):e0201706. doi: 10.1371/journal.pone.0201706 (PMC6128456; doi:10.1371/journal.pone.0201706)

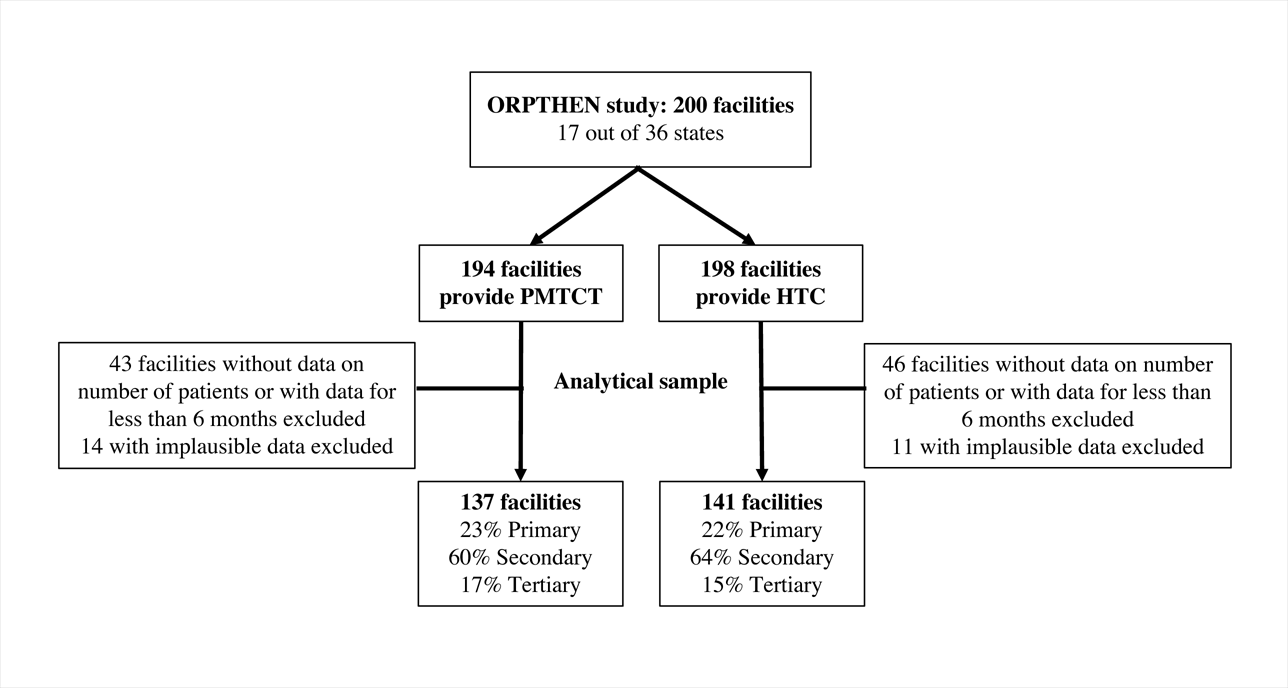

Supplement: S1 Fig — (TIF) [file pone.0201706.s002.tif]

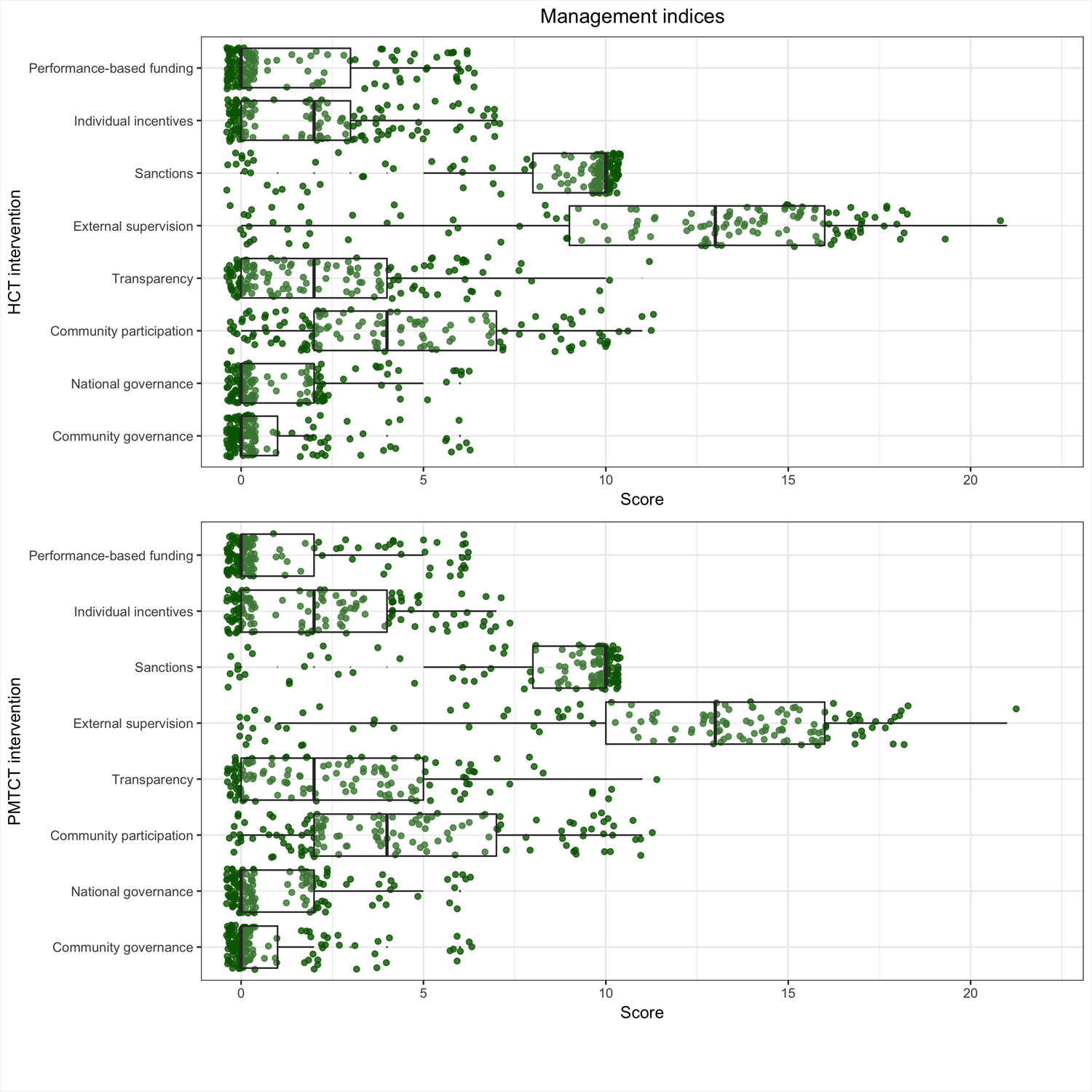

Supplement: S2 Fig — (TIF) [file pone.0201706.s003.tif]
